# Doppler shift compensation performance in *Hipposideros pratti* across experimental paradigms

**DOI:** 10.3389/fnsys.2022.920703

**Published:** 2022-08-01

**Authors:** Jinhong Luo, Manman Lu, Xindong Wang, Huimin Wang, Cynthia F. Moss

**Affiliations:** ^1^School of Life Sciences, Institute of Evolution and Ecology, Central China Normal University, Wuhan, China; ^2^Department of Psychological and Brain Sciences, Johns Hopkins University, Baltimore, MD, United States

**Keywords:** auditory feedback, behavioral plasticity, echolocation, motor control, neuroethology

## Abstract

A central aim of neuroethological research is to discover the mechanisms of natural behaviors in controlled laboratory studies. This goal, however, comes with challenges, namely the selection of experimental paradigms that allow full expression of natural behaviors. Here, we explore this problem in echolocating bats that evolved Doppler shift compensation (DSC) of sonar vocalizations to yield close matching between echo frequency and hearing sensitivity. We ask if behavioral tasks influence the precision of DSC in Pratt’s roundleaf bat, *Hipposideros pratti*, in three classic laboratory paradigms evoking audio-vocal adjustments: Stationary bats listening to echo playbacks, bats transported on a moving pendulum, and bats flying freely. We found that experimental conditions had a strong influence on the expression of the audiovocal frequency adjustments in bats. *H. pratti* exhibited robust DSC in both free-flying and moving-pendulum experiments but did not exhibit consistent audiovocal adjustments in echo playback experiments. *H. pratti* featured a maximum compensation magnitude of 87% and a compensation precision of 0.27% in the free flight experiment. Interestingly, in the moving pendulum experiment *H. pratti* displayed surprisingly high-precision DSC, with an 84% maximum compensation magnitude and a 0.27% compensation precision. Such DSC performance places *H. pratti* among the bat species exhibiting the most precise audio-vocal control of echo frequency. These data support the emerging view that Hipposiderid bats have a high-precision DSC system and highlight the importance of selecting experimental paradigms that yield the expression of robust natural behaviors.

## Introduction

Controlled laboratory studies are employed to discover the mechanisms of natural animal behaviors. Laboratory settings allow researchers to experimentally study selected behaviors, while controlling for environmental variables, offering an opportunity to discover mechanisms that would otherwise prove difficult to reveal in an organism’s natural environment. However, studying animals in a laboratory setting comes with other challenges or potential pitfalls. The housing environment alone, for example, can impact many aspects of animal behavior and physiology, including reproduction, circadian rhythm, and immune function, just to list a few (Calisi and Bentley, 2009). Particularly noteworthy, some observations in laboratory experiments may differ from those in the natural environment (Kronfeld-Schor et al., 2013). Hence, understanding the effects of controlled laboratory settings on animals’ expression of natural behavior is of great importance.

Echolocating bats are a choice animal model for neuroethological research, partly due to their active sensing behaviors, which can be quantitatively analyzed and linked to neural processes (Griffin, 1958; Moss et al., 2011; Luo and Moss, 2017; Luo et al., 2017, 2018; Kothari et al., 2018). Studying echolocating bats in controlled laboratory environments dates back to Spallanzani’s question of how bats avoid obstacles in the dark, as well as to the groundbreaking observations by Donald Griffin and Robert Galambos (Griffin and Galambos, 1941; Griffin, 1958; Grinnell, 2018). Since then, controlled laboratory settings have continued to unravel the mechanisms of echolocation in bats (Popper and Fay, 1995; Thomas et al., 2004; Fenton et al., 2016). In recent years, however, several studies have pointed to distinct differences in the echolocation behavior of bats in the laboratory and in the field. For example, the big brown bat, *Eptesicus fuscus*, does not produce long duration search calls in the laboratory, as it does in the field (Surlykke and Moss, 2000). Daubenton’s bat, *Myotis daubentonii*, emits more directional calls of higher intensity in the field than in the laboratory (Surlykke et al., 2009). The minimum frequency of the first search call emitted after the buzz phase in *M. daubentonii* also shows differences between successful and unsuccessful prey captures, but only in the laboratory, and not in the field (Britton and Jones, 1999).

**FIGURE 1:**
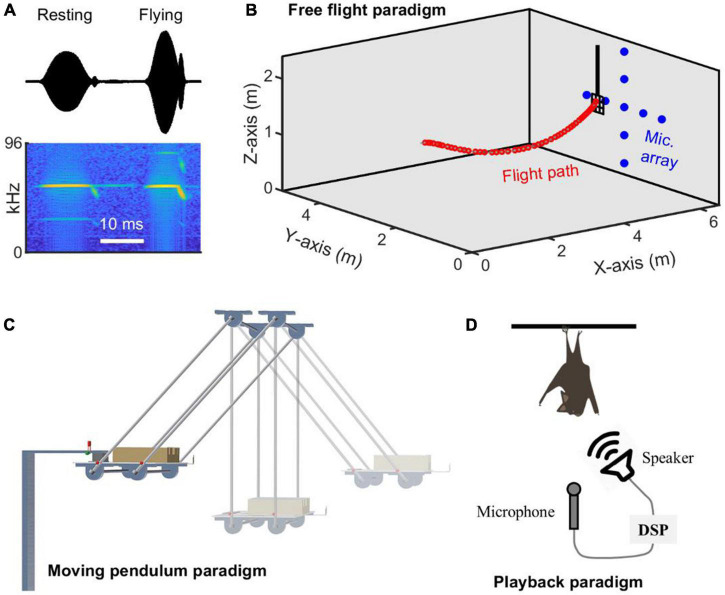
Three typical experimental paradigms employed for quantifying Doppler shift compensation behavior in CF-FM echolocating bats. **(A)** Typical examples of echolocation calls produced by the CF-FM bat *Hipposideros pratti* when hanging freely and flying toward a landing platform. Note that the duration, as well as the energy distribution across harmonics, are different, with the call from the flying situation shorter in duration, weaker, and stronger in energy for the first and third harmonics, respectively. **(B)** Free flight paradigm, in which echolocation calls of a trained flying bat were recorded by an array of microphones mounted on the wall, and the flight path was reconstructed based on microphone recordings. **(C)** Moving pendulum paradigm, in which a body-restrained bat was swung back and forth, and the echolocation calls were recorded by an onboard microphone in front of the bat’s mouth. **(D)** Playback paradigm, in which a hanging (resting) bat spontaneously produces echolocation calls and receives a frequency-shifted copy of the calls online. In turn, bats may adjust the call frequency in response to frequency-altered auditory feedback. DSP, digital signal processor.

In laboratory settings, experimental paradigms can also affect the expression of bats’ natural echolocation behaviors, such as Doppler shift compensation (DSC). DSC is found in bat species that produce echolocation calls consisting of relatively long constant-frequency (CF) components, in combination with frequency modulated (FM) components, and these species are commonly referred to as CF-FM bats (Figure 1A). Bats exhibiting DSC are found in the families of Rhinolophidae and Hipposideridae, and two species of Mormoopidae (*Pteronotus parnellii* and *P. personatus*) (Smotherman and Guillén-Servent, 2008; Schnitzler and Denzinger, 2011). Two species of Noctilionidae (*Noctilio albiventris*; *N. leporinus*) that produce quasi-CF signals exhibit partial DSC (Wenstrup and Suthers, 1984). During flight, the echoes received by bats as they approach targets are up-shifted in frequency, due to the Doppler effect, and CF-FM bats show DSC behavior by lowering the emitted call frequency so that the echo frequency is maintained in a narrow frequency range of the bat’s most sensitive hearing (Schnitzler, 1968, 1973; Schnitzler and Denzinger, 2011; Hiryu et al., 2016). DSC is one of the most intensely studied audio-vocal behaviors in echolocating bats, and three experimental paradigms have been widely used to investigate the details of their feedback control (Figures 1B–D). Although qualitative differences in DSC experimental paradigms have been anecdotally mentioned, most published work has only reported data from a single experimental method (e.g., Schnitzler and Denzinger, 2011; Hiryu et al., 2016). These qualitative comparisons suggested that all flying bats exhibit robust DSC behavior, while bats swung on a pendulum or performing in playback experiments tended to show reduced DSC.

In particular, the expression of DSC behavior in Hipposiderid bats seems to be very sensitive to experimental paradigms. For both *H. speoris* and *H. bicolor*, DSC behavior was not observed in a playback experiment (Schuller, 1980), but partial DSC behavior, with a maximum compensation magnitude of about 55%, was observed in bats moving on a pendulum (Habersetzer et al., 1984). By contrast, two recent studies of *H. armiger* in free-flying experiments reported precision of DSC (0.15∼0.17%) (Schoeppler et al., 2018; Zhang et al., 2019), much higher than previous reports of 0.4∼0.7% (Schnitzler and Denzinger, 2011). Compensation precision is measured as the percentage ratio of the standard variation to the mean of the echo frequencies. The smaller the value, the higher the compensation precision. Furthermore, two studies quantified and compared the DSC performance of *P. parnellii* between two experimental paradigms and confirmed that the accuracy of DSC in *P. parnellii* was indeed higher in free-flying bats than in animals swung on a pendulum (Lancaster et al., 1992; Keating et al., 1994). Nevertheless, one advantage of the playback and moving pendulum paradigms is that the experimental subject remains at the same position during the test, which not only reduces technical challenges of neurophysiological investigations, but also allows for isolating experimental variables that may covary in free-flying experiments. It is noteworthy that both playback and moving pendulum paradigms have also been used to study audiovocal control capability of bat species that do not exhibit DSC behavior, such as the big brown bat (Luo and Moss, 2017) and the Seba’s short-tailed bat (Beetz et al., 2021).

Considering the technical advantages of playback and pendulum paradigms for probing mechanisms of DSC behavior and the long under-appreciated high accuracy of DSC in Hipposiderid bats, we measured and quantitatively compared the DSC behavior in *Hipposideros pratti* across the three experimental paradigms. Our data show that *H. pratti* did not exhibit consistent audiovocal adjustments in the playback experiment, but exhibited robust DSC behavior in both free flight and moving pendulum experiments, with an overall compensation precision in these two paradigms of 0.27%.

## Results

We conducted behavioral experiments using the CF-FM bat, *H. pratti*, in the laboratory and compared DSC performance in three experimental paradigms (Figures 1B–D), including bats trained to fly and land on a platform, bats transported in a moving pendulum, and hanging bats listening to frequency-shifted playbacks of their echolocation calls.

### Overall vocal behavior of *Hipposideros pratti* in three experimental paradigms

#### Free flight paradigm

**FIGURE 2:**
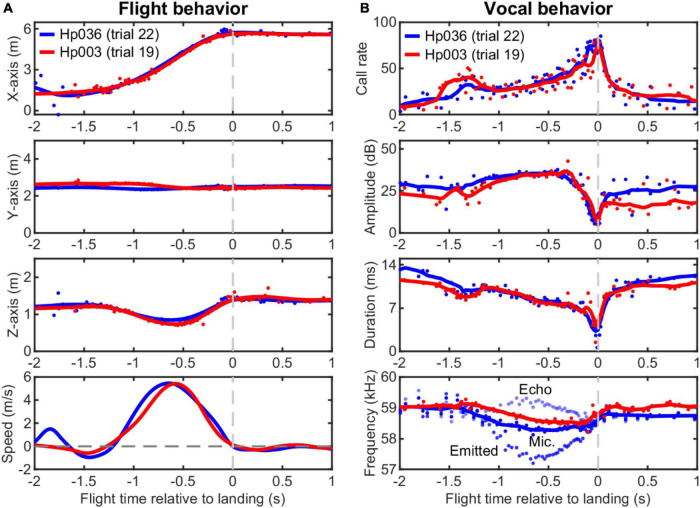
Example flight and echolocation behavior of two individuals of *Hipposideros pratti* trained to approach and land on a platform in a flight room. **(A)** Reconstructed three-dimensional (3D) spatial position and the estimated flight speed of the bats using a nine-microphone array mounted on the wall. **(B)** Dynamic adjustments of call parameters of the bats during the approaching flight. Note the highly consistent vocal pattern of the bats for the adjustments of call rate, call amplitude, and call duration that can signify the time of landing events. The call amplitude was measured directly from the central microphone of the array without extra compensation. Based on flight speed (relative to the microphone wall) and call frequency recorded by the static (ground) microphones, the emitted call frequency, and the received echo frequency can be estimated. Thus, we assumed that here that the bat performed DSC using echoes from the microphone wall to its front. For clarity, here we only plotted the emitted call frequency and echo frequency for Hp036. For all panels, original data points are shown as circles and the smoothed traces (nine-point moving average) are shown as solid lines.

Four individual *H. pratti* (two males and two females) were successfully trained to start from an elevated position, fly toward, and land on a hanging grid (20 cm × 20 cm) over a distance of approximately 4 m in the laboratory (Figure 1B). After each successful landing, the bat received a piece of food reward. For each flight trial, echolocation calls of the bat were recorded by an array of nine broadband ultrasound microphones mounted on the wall facing the approaching animal. All bats learned to perform the landing task after approximately 1 month of training, but data collection only started after bats have been trained for approximately 2 months. *H. pratti* exhibited stereotypical flight behavior, with trajectories typically straight toward the landing platform. Two example flight trials from two individuals are shown in Figure 2A. The 3D spatial positions of *H. pratti* at the time of call emission were reconstructed using the time of arrival differences from the microphone array. From the reconstructed 3D spatial positions between two consecutive calls, we estimated the instantaneous flight speeds of the bats.

*Hipposideros pratti* exhibited dynamic vocal behavior during an approach to the landing platform (Figure 2B). Specifically, *H. pratti* reached a maximum call rate, a minimum call amplitude, and a minimum call duration around the time of landing. In this study, we used the median of the three time estimates when *H. pratti* reached a maximum call rate, a minimum call amplitude, and a minimum call duration to represent the landing time. This “vocal” landing time may be slightly different from the landing time when they touched the platform, which was not measured. Nevertheless, it is interesting to note that the landing times decoded independently from the three vocal parameters were highly similar and indistinguishable statistically (Paired non-parametric sign-rank test, all three *P* > 0.78). The recorded calls by the static microphones on the wall indicate that *H. pratti* decreased call frequency (peak frequency of the CF component for the dominant 2nd harmonic) during flight, a manifestation of the DSC behavior. Figure 2B (bottom panel) shows the emitted call frequency and the received echo frequency of a typical trial.

#### Moving pendulum paradigm

**FIGURE 3:**
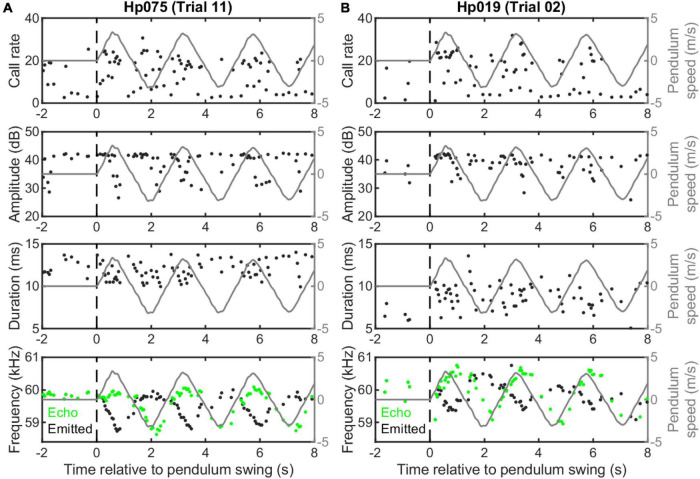
Example echolocation behavior of two individuals of *Hipposideros pratti* swung on a pendulum setup. Two trials illustrating higher **(A)** and lower **(B)** quality Doppler shift compensation (DSC) performance of the two bats, as judged by the compensation magnitude and echo-frequency variation. Note, both bats compensated for the Doppler effect by lowering the emitted call frequency only for the forward, but not the backward swings. Also, there were no clear cyclic adjustments of call rate, call amplitude, or call duration during the swings.

We built a moving pendulum setup that consists of a bat holder to restrain the body of *H. pratti* and a miniature microphone (6 mm × 10 mm), mounted in front of the bat nose, to record echolocation calls (Figure 1C). For each trial, the pendulum carrying a bat was released from an elevated position (approximately 45° relative to freely hanging pendulum) with an electromagnetic switch and swung toward a reflective whiteboard (2.2 m × 1.5 m). The trajectory of the moving pendulum was recorded with a high-speed video camera at 100 fps, from which the spatial position and speed of the pendulum were estimated. The distance between the whiteboard and the freely hanging pendulum was 1.5 m, and the minimum and maximum distances between the bat and the whiteboard were 0.2 and 2.56 m. Thus, the bat received high-amplitude echoes at relatively short delays between 1.2 and 15.1 ms. The pendulum reached a maximum speed of 3.34 ± 0.09 m/s and featured a cyclic period of 2.56 ± 0.03 s (Figure 3).

We did not find cyclic vocal adjustments in call rate, amplitude, or duration, but found clear DSC behavior in *H. pratti* (Figure 3). It is noteworthy that *H. pratti* tended to produce calls of reduced amplitude and duration during the second half of the forward swings. Similar to findings observed for other CF-FM bat species in a moving pendulum setup (Habersetzer et al., 1984; Gaioni et al., 1990; Behrend and Schuller, 1999; Boonman et al., 2020), *H. pratti* only compensated for an increase in echo frequency during forward swings by decreasing the emitted call frequency, but not for a decrease in echo frequency during backward swings. Figure 3 (bottom panels) shows two example trials, in which the maximum frequency decreases for two individuals were 1.11 and 0.67 kHz, representing a maximum compensation percentage of 98.6 and 62.8%. The compensation precisions in these two trials, measured as the percentage ratio of the standard variation to the mean of the echo frequencies during the forward swing, were 0.18 and 0.42%. As these two trials were selected to show the maximum variability of the DSC performance of *H. pratti* in the moving pendulum paradigm, it seems that *H. pratti* generally exhibited robust DSC behavior when swinging on a pendulum.

#### Real-time playback paradigm

**FIGURE 4:**
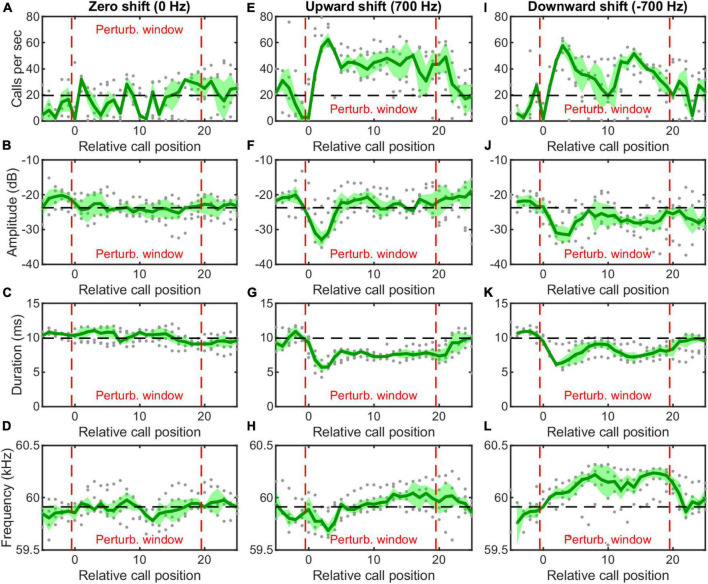
Vocal behavior of a hanging *Hipposideros pratti* in response to online frequency-shifted auditory feedback. For each trial, the bat received 20 consecutive frequency-shifted auditory feedback during the perturbation window (between the red dashed lines). The bat did not make any vocal adjustment when received 0-Hz shifted auditory feedback **(A–D)**, but increased call rate **(E,I)**, decreased call amplitude **(F,J)** and call duration **(G,K)**, and decreased **(H)** or increased **(L)** call frequency when received upward shifted and downward shifted auditory feedback for at least a few vocalizations. The gray dots show the individual trial data; the green line and the shaded green area show the average and standard deviation for all the trials (five trials per condition and bat).

We used an auditory feedback perturbation system to broadcast frequency-shifted copies of resting bats’ vocalizations at a short time delay. For each trial, one hanging *H. pratti* received 20 consecutive frequency-shifted echolocation calls of a predetermined shift size and delay (Figure 1D). In total, we made preliminary recordings (five trials per bat per condition) from four individual *H. pratti*. Figure 4 showed vocal behaviors of one *H. pratti* from three feedback conditions of 0, 700, and –700 Hz shift sizes. The perturbation window is surrounded by two red dashed vertical lines. We found that during the perturbation window the bat did not adjust either call rate, call amplitude, call duration, or call frequency (peak CF) in response to 0-Hz shifted feedback stimuli (Figures 4A–D). By contrast, in response to both 700 and –700 Hz frequency shifted stimuli, the bat increased call rate (Figures 4E,I), decreased call amplitude (Figures 4F,J) and call duration (Figures 4G,K), at least for the first few calls during the perturbation window. Interestingly, the bat not only decreased the peak frequency for the first a few calls in response to 700 Hz frequency shifted stimuli, but also increased the peak frequency in response to –700 Hz frequency shifted stimuli. That is, the bat exhibited bidirectional compensatory frequency adjustments to auditory feedback stimuli, which is thus not consistent with a DSC behavior in freely flying bats (Figure 2B, bottom panel) or bats transported in a moving pendulum setup (Figure 3, bottom panels). In a detailed study with the same playback setup on another Hipposiderid bat, *H. armiger*, we have shown that online vocal frequency adjustments by *H. armiger* are driven by sensory prediction errors, but not by DSC or by jamming avoidance response (Wang et al., 2022). Thus, below we only made detailed statistical comparisons of the DSC performance of *H. pratti* in the free flight and moving pendulum paradigms.

### A comparison of Doppler shift compensation performance between free flight and moving pendulum paradigms

**FIGURE 5:**
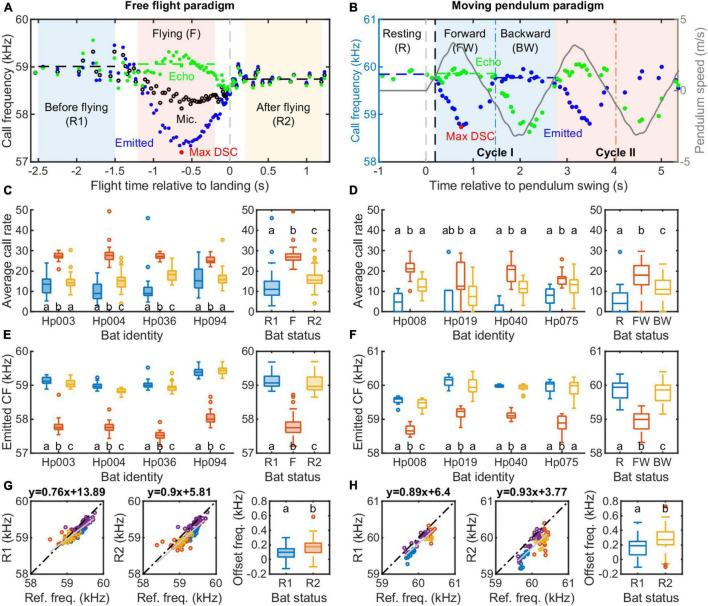
Vocal behavior of *Hipposideros pratti* in the free flight and moving pendulum paradigms. **(A)** A detailed illustration of the DSC behavior of a flying bat. For quantitative analyses, a flying trial was divided into three one-second sections: before flying (R1; –2.5∼–1.5 s), during flying (F, –1.2∼–0.2 s), and after flying (R2; 0.2∼1.2 s). Time was referred to as the median of the landing time estimated by the vocal behavior when the bat reached the maximum call rate, minimum call amplitude, and call duration (Figure 2B; see section “Materials and methods” for more details). **(B)** A detailed illustration of the DSC behavior of a restrained bat on a moving pendulum. Similar to the flying trials, a pendulum trial was dived into three sections: resting (R, –1∼0 s), Forward (FW) swing, and Backward (BW) swing. For the forward and backward swings, the start and end times were adjusted by the vocal reaction time (delay) of the bat in each trial. Average call rate **(C)** and call frequency **(E)** of *H. pratti* before, during, and after the approach flight at the individual and species levels. Average call rate **(D)** and call frequency **(F)** of *Hipposideros pratti* before (resting), during the forward, and backward swings at the individual and species levels. **(G)** Correlations between the reference frequency and two types of resting frequency (R1, before flying; R2, after flying), and a comparison of the offset frequency between R1 and R2 for the free-flying bats. **(H)** Correlations between the reference frequency and resting frequency (before swing onset) and call frequency during the backward swing, and a comparison of the offset frequency between resting and backward swing for the moving pendulum bats. The sample size for the four individuals, i.e., the number of trials, ranged from 34 to 36 in the free flight experiment, and from 18 to 20 in the moving pendulum experiment.

Before we can directly compare the DSC performance of *H. pratti* between the free flight and moving pendulum paradigms, several methodological details should be explained, due to their inherent differences. One principle parameter commonly used to evaluate DSC performance in CF-FM bats is compensation magnitude. Compensation magnitude is typically estimated as the percentage of the maximum frequency reduction by a bat to the speed-induced or Doppler-effect-induced change in resting frequency. Thus, a 100% (full) compensation magnitude means that the CF echo frequency received by the bat during flight, also referred to as reference frequency, equals the CF resting frequency produced by a stationary bat. It is widely reported that the reference frequency of flying CF-FM bats is slightly higher than its resting frequency, with a difference of ∼150–200 Hz in Rhinolophids and *P. parnellii* (Schnitzler and Denzinger, 2011). This means that CF-FM bats under-compensate for the Doppler effect and actively maintain a small frequency offset. Thus, estimating the compensation magnitude requires estimations of the resting frequency and the flight speed. Although most studies quantify resting frequency as the average call frequency before bats launch into flight (Schnitzler, 1973; Schoeppler et al., 2018), some studies report the average call frequency measured in bats after landing (Hiryu et al., 2008). In this study, we analyzed resting frequency both before the bat took off and after the bat landed on the platform (Figure 5A, R1 and R2 phases). Since we did not measure the precise flight onset and offset time with video, and the estimated flight speeds were estimated from microphone array recordings, we took a conservative approach and characterize the following phases: –2.5∼–1.5 s resting before flying (R1), –1.2∼–0.2 s while flying (F), and 0.2∼1.2 s and resting after flying (R2). Time 0 is the vocal landing time. Another reason to exclude the data shortly before the landing is that during this critical period the bat rotates its body and head from a flight posture to upside-down posture. The bats probably do not rely on the echoes from the microphone wall for DSC during this manuever. Compensation precision was estimated from the flying phase (F). Similar to other bat species transported in a moving pendulum setup (Gaioni et al., 1990; Boonman et al., 2020), there is a reaction time for *H. pratti* in initiating the DSC. In the example trial shown in Figure 5B, the reaction time was 0.195 s. We accounted for the reaction time when defining the Resting (R), Forward (FW), and Backward (BW) swing phases. DSC precision was quantified for the forward swing.

The average call rate of freely flying *H. pratti* in the R1, flying (F), and R2 phases are presented in Figure 5C; the average call rate of pendulum-transported *H. pratti* in the R, FW, and BW phases are presented in Figure 5D. All individuals of *H. pratti* show the highest average call rate during the flying (F) phase and the forward swing phase in the free-flying experiment and the pendulum experiment, respectively (Figures 5C,D). Similarly, all individuals of *H. pratti* emitted the lowest call frequency during the flying (F) phase and the forward swing phase of the free flight experiment and the pendulum experiment, respectively (Figures 5E,F). Figures 5G,H show the relationship between the reference frequency and the resting frequencies. We found that for both experimental paradigms, resting frequency estimated from the “post-flight” phase, i.e., after flying (R2) and during the backward swing (BW), showed a more linear relationship with the reference frequency (Slope: 0.9 vs. 0.76; and 0.93 vs. 0.89). The offset frequency was larger when estimated from the “post-flight” (R2 or BW) phase than from the “pre-flight” (R1 or R) phase (Median: 100 vs. 174 Hz and 188 vs. 272 Hz).

**FIGURE 6:**
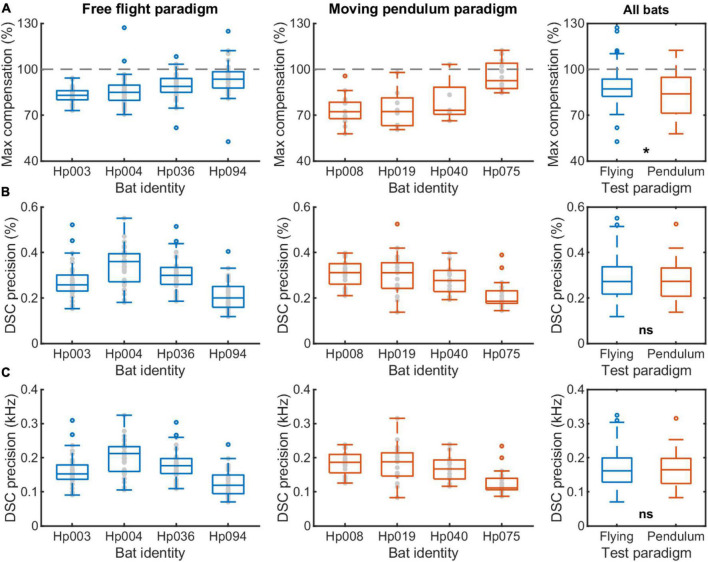
A comparison of the DSC performance of *Hipposideros pratti* between the free flight and moving pendulum paradigms. **(A)** Maximum compensation magnitude in percentage. 100% indicates a full compensation for the flight speed induced echo frequency change due to the Doppler effect based on the resting frequency before flying or pendulum swing onset. **(B)** DSC precision in percentage, which was the ratio between the standard variation to the average of the echo frequencies during the forward swing. **(C)** DSC precision in kHz, which was the standard variation of the echo frequencies during the forward swing. The sample size for the four individuals, i.e., the number of trials, ranged from 34 to 36 in the free flight experiment, and from 18 to 20 in the moving pendulum experiment.

Next, we compared the maximum compensation magnitude and compensation precision between the free flight and moving pendulum paradigms (Figure 6). We found that overall *H. pratti* in the free flight experiment had a slightly higher compensation magnitude than in the moving pendulum experiment (Figure 6A; Medians, 87.2 vs. 83.9%; Non-parametric Rank-sum test, *P* < 0.05). This difference can also be seen in the offset frequency that was about 100 Hz larger in the moving pendulum experiment (Figures 5G,H). However, *H. pratti* exhibited similar compensation precision in both experimental paradigms (Figures 6B,C). The overall median compensation precision of *H. pratti* was 0.27 and 0.27% (Figure 6B; Non-parametric Rank-sum test, *P* > 0.05), which corresponding to variability (standard variation) in echo frequency of 162 and 165 Hz, respectively (Figure 6C; Non-parametric Rank-sum test, *P* > 0.05). As a reference, the median variability of resting frequency before and after flying phases of *H. pratti* in the free flight paradigm was 118 Hz (Quartiles: 65 and 136 Hz) and 116 Hz (Quartiles: 101 and 134 Hz); the median variability of resting frequency before and during the backward swing phases of *H. pratti* in the moving pendulum paradigm was 107 Hz (Quartiles: 67 and 128 Hz) and 149 Hz (Quartiles: 117 and 207 Hz).

## Discussion

Compared to the greater horseshoe bats (*R. ferrumequinum*) and mustached bats (*P. parnellii*), less research effort has been devoted to quantifying DSC performance in Hipposiderid bats. It was long believed that Hipposiderid bats are not able to adjust CF call frequencies to accurately stabilize echo frequencies (Schnitzler and Denzinger, 2011; Hiryu et al., 2016), a view that has been refuted recently with data from *H. armiger* in flight (Schoeppler et al., 2018; Zhang et al., 2019). These two recent studies showed that *H. armiger* in flight shows an overall DSC precision of 0.15∼0.17%, which is comparable to or only slightly poorer than the 0.1∼0.2% compensation precision reported for Horseshoe bats (*R. ferrumequinum, R. euryale, R. rouxii*) and *P. parnellii* (Schnitzler and Denzinger, 2011; Zhang et al., 2019). Our study reveals that *H. pratti* in flight exhibits an overall compensation precision of 0.27%, or a 160 Hz standard variation of the echo frequencies, supporting the emerging view that Hipposiderid bats indeed feature a high-precision DSC system.

Studies of Rhinolophid bats suggest that there are no differences in compensation precision among the species tested in flight, and all featured a compensation precision of 0.1–0.2% (Schnitzler and Denzinger, 2011). However, our data suggest that the precision of the DSC system may be species-specific within the same genus of Hipposiderid bats, with 0.27% compensation precision of *H. pratti*, as reported in the current study, compared with 0.15∼0.17% compensation precision of *H. armiger* (Schoeppler et al., 2018; Zhang et al., 2019). *H. pratti* emits CF echolocation signals that are approximately 12 kHz lower in frequency than *H. armiger* (Lu et al., 2020). Because compensation precision is computed as the percentage ratio of the standard deviation to the mean of the reference frequency, the same value of standard deviation would result in a larger estimate of DSC precision when the reference frequency is lower. Can the 12 kHz difference between *H. pratti* and *H. armiger* account for the observed DSC precision in these two species? Simple math suggests that this is not the case. The ∼12 kHz reference frequency difference can only account for approximately 4% of the compensation precision differences between species. The compensation precision, when measured as a standard deviation around the reference frequency, is still 55 Hz more variable in *H. pratti* than in *H. armiger*. However, a caveat in the precision estimate of *H. pratti* in the current study concerns its flight speed. The study of DSC behavior in flying bats is typically determined from synchronized high-speed video recordings; however, in the current study, we localized the flying bat and derived the instantaneous flight speed using measurements taken with a microphone array. It is known that acoustic localization of sound sources is less accurate than optical methods, with an error up to 5% of the distance to the sound source for some bat species (Surlykke and Kalko, 2008). Thus, our acoustic localization method adds error in the estimation of the bat’s instantaneous position and the computed flight speed, which affects the compensation precision. Thus, in future work, it will be critical to measure and compare the compensation precision of *H. pratti* with more accurate localization systems, such as high-speed video, to test whether the compensation precision is species-specific in Hipposideros bats.

One unexpected result from the current study is that *H. pratti* exhibited precise DSC behavior in the moving pendulum experiment. Specifically, the overall compensation precision in four *H. pratti* was 0.27%, ranging from 0.19 to 0.31% across individual animals. The overall maximum compensation magnitude of *H. pratti* was 83.9%, ranging from 72.3 to 92.6% across individual animals. An overall compensation precision of 0.27% is more precise than the overall 0.43% compensation precision reported for *P. parnellii* in a moving pendulum experiment (Lancaster et al., 1992). One reason for a relatively large value of the compensation precision of *P. parnellii* in the study by Lancaster et al. (1992) is that the authors may not have corrected for the bat’s reaction time, as pointed out in a study of *P. parnellii* tested with a moving pendulum setup (Gaioni et al., 1990). The study by Gaioni et al. (1990), however, did not report the compensation precision. In our study, we not only corrected for the reaction time of the bat in each trial, but also used an optimization method to search for the reaction time that returned the highest compensation precision. Any other methods, such as the one used by Gaioni et al. (1990), in which reaction time was measured as the time delay between the maximum pendulum speed and the lowest call frequency of the bat, would result in a lower compensation precision than our method.

We found that *H. pratti* compensated for an overall 84% of full frequency shifts in the moving pendulum experiment, which is among the greatest compensation of bat species tested on swinging pendulums. A previous study reported an overall maximum compensation magnitude of 55 and 56% in two other species of Hipposiderid bat, *H. speoris* and *H. bicolor*, respectively in a moving pendulum experiment (Habersetzer et al., 1984). *P. parnellii* was reported to compensate for on average 80% of the Doppler-shifted echoes (Gaioni et al., 1990). Boonman et al. (2020) found an overall compensation magnitude generally below 80% for three species of Horseshoe bat, *R. ferrumequinum*, *R. blasii*, and *R. hipposideros* (Boonman et al., 2020). It is noteworthy that *R. rouxii* was considered to show a “full compensation” in a pendulum setup as this species’ echo frequency fell within a ±300 Hz window around the resting frequency, yet quantitative measurements were not reported (Behrend and Schuller, 1999). Playback experiments on *R. ferrumequinum* suggest that the compensation magnitude of the DSC behavior can be affected by several properties of returning echoes. *R. ferrumequinum*, for example, exhibits a reduced compensation magnitude with decreasing echo amplitude (Smotherman and Metzner, 2003) and with increasing echo delay (Schuller, 1974). Moreover, a higher rate of call emissions also leads to a higher compensation magnitude in *R. ferrumequinum* (Schuller, 1986). In our study, we placed a highly reflective wall at a short distance from the pendulum, which returns high-amplitude echoes at short delays. Bats also significantly increased call rate during the forward swings, compared with both the resting phase and backward swings (Figure 5D). Thus, this scenario of echo feedback may have yielded favorable conditions to induce greater DSC magnitude in *H. pratti*. Furthermore, we suggest that the slightly higher average call rate of *H. pratti* in the free flight experiment than in the moving pendulum experiment, as shown in Figures 5C,D, may also account for the 3% higher maximum compensation magnitude in the freely flying bats (Figure 6A).

To conclude, we have shown that a moving pendulum setup offers a suitable experimental paradigm to investigate the DSC performance of *H. pratti*, and this method offers advantages for concurrent neurophysiological recordings of behaving bats. Our data support the emerging view that Hipposiderid bats have a high-precision DSC system. Methodological inaccuracy in estimating the reaction time that is required to evaluate the DSC performance of bats in pendulum experiments may partially account for an underestimated compensation precision reported in previous studies. It is noteworthy that *H. pratti* in the moving pendulum and free flight experiment exhibited similar DSC performance, but did not show consistent audiovocal adjustments in the playback experiment. Nevertheless, it should be noted that the DSC behavior of bats in the moving pendulum experiment differs from behavior in the free flight experiment in several critical aspects, including (1) there is a clear reaction time of bats in the moving pendulum experiment, but not in the free flight experiment; (2) the average call rate of *H. pratti* is significantly lower in the pendulum experiment than in the free flight experiment, across the resting phases and flying phase; (3) while DSC behavior is accompanied by adjustments of several other signal parameters, such as call amplitude and duration in the free flight experiment, linked vocal adjustments are largely lacking in the moving pendulum experiment. What factors cause these differences will be the subject of future studies.

## Materials and methods

### Animals

In each of three experiments, four adult *H. pratti* were tested for DSC. The sex of the animals were two males and two females for the free flight experiment, four males for the pendulum experiment, and two males and two females for the playback experiment. Yet, we did not specifically select the sex of the animals in either experiment. All bats were wild-caught with hand nets during the daytime in a cave of Xianning City, Hubei province, China. Bats were housed in social groups of two to five, in custom-made metal cages (40 cm × 40 cm × 40 cm), placed in a room with a regulated air temperature of around 24°C, relative humidity of around 60%, and a reversed light regime of 12 h darkness and 12 h light. Bats had *ad libitum* access to water and food. Capture, housing, and behavioral studies were approved by the Institutional Animal Care and Use Committee of the Central China Normal University.

### Experimental setups

All three experiments were conducted in the same test room (6.5 m × 5 m × 2.3 m, length × width × height). The walls and ceiling of the room were covered with acoustic foam of an 8 cm thickness, and the floor was covered with nylon blankets to reduce the echo reflections. In the free flight experiment (Figure 1B), a landing grid (20 cm × 20 cm) hung about 0.9 m from the ceiling at approximately 0.8 m distance from a wall equipped with a microphone array. The microphone array contained nine broadband ultrasound microphones (NEUmic, Ultra Sound Advice, United Kingdom) that were configurated into a “+” shape. All microphones were fixed toward the opposite wall in the direction of the approaching bat and the microphone-to-microphone distance was approximately 0.5 m. Note, the exact three-dimensional (3D) position of each microphone was accurately determined with a 1 cm precision at least with a ruler (1 mm precision) for reconstructing the flight path of the vocalizing bat. For each trial, the bat was released from a raised hand of an experimenter from a position close to the pendulum (see below) and flew approximately 4 m to land on the suspended grid. Although all four *H. pratti* participating in the flying experiment learned to perform the landing task after approximately 1 month of training, data collection for this experiment only started after bats have been trained for approximately 2 months. Training generally took place 5–6 days per week.

For the moving pendulum experiment, a pendulum (Figure 1C) was attached to the ceiling close to the wall opposite the microphone array. The pendulum, with an arm length of 1.75 m, was located at 1.6, 2.5, and 2.1 m to the back, left and right wall, and 0.6 m above the floor of the test room, when hanging freely. A reflective wooden board was placed 1.5 m in front of the free-hanging pendulum to return high-intensity echoes to the bat at short delays. The pendulum consisted of a bat holder to restrain the body, but allowed free movement of the head of *H. pratti*. The bat holder was made of metal frames filled with foam in which *H. pratti* maintained a crawling posture. In front of the bat nose, at a distance of 7 cm, a miniature microphone (Custom made, based on SPU0410LR5H, Knowles Corporation, Itasca, IL, United States) was attached to the bat holder frame with an “L” shaped metal bar. Thus, the microphone swung together with the bat in the pendulum and recorded emitted calls without the Doppler effect. Two illuminated colored LEDs separated by 15.3 cm were fixed to one side of the bat holder to facilitate video tracking of the bat’s position during the swings by a video camera (1920 × 1080 quality at a100 frame rate; Model FDR-AX700, SONY, Japan). The pendulum was pulled toward the back wall and attached to an electromagnetic switch before the start of each trial. The pendulum movement was started by cutting off the power of the magnetic switch. Video recording and microphone recording were synchronized through a third LED that was lighted by a voltage signal output from an audio interface (see section “Sound recording”). The same voltage signal was recorded by a microphone channel *via* a shortcut cable, while the camera detected the LED signal. The accuracy of the synchronization was ∼10 ms, which was constrained by the much lower sampling rate of the camera (100 Hz), compared to the sampling rate of sound recording (192 kHz).

During the playback experiment, an *H. pratti* hung freely on an elevated (2 m) platform attached to a tripod standing on the floor (Figure 1D). The playback setup consisted of a measurement microphone (7016, 1/4-inch Condenser microphone, ACO Pacific, Belmont, CA, United States; with protection grid on) and an ultrasound loudspeaker (Vifa, Avisoft Bioacoustics, Berlin, Germany), which were placed at a 20 and 15 cm distance, respectively, in front of the bat, with the loudspeaker about 15° off the midline. Hanging *H. pratti* produced echolocation calls spontaneously in the setup and received frequency-shifted echo simulating objects at a short delay of ∼4 ms, including 0.6 ms delay for signal processing, 1 ms delay for signal transmissions, and 2 ms digital delay introduced by the experimenter. In this study, we tested bats with three frequency shift sizes of 0, 700, and –700 Hz. A 700 Hz positive frequency shift would be experienced by *H. pratti* flying at a speed of 2 m/s. Similar to other Hipposiderid bats, flying *H. pratti* lower their call frequency to compensate for a positive frequency shift (Figure 2B, bottom panel). The amplitude of the echo playbacks was approximately 15 dB weaker than the emitted call, with the maximum peak amplitude of the echo playback at approximately 90 dB SPL. Note, in addition to the echo playbacks, *H. pratti* also received echoes from nearby physical objects, such as the microphone, the loudspeaker, and the floor and walls. A detailed description of the playback setup has been described in a study of *H. armiger* (Under review).

### Sound recording

In both the flying and pendulum experiments, echolocation calls of *H. pratti* were recorded, amplified, and digitized before being saved to the hard drive of a desktop computer. In the flying bat experiment, the microphone signal was amplitude by its internal amplifier (i.e., NEUmic); in the pendulum experiment, the microphone signal was amplified by the audio interface amplifier (Fireface 802, RME, Germany). The same audio interface was used to convert analog microphone signals into digital signals at a sampling rate of 192 kHz. Setups were controlled through custom-written programs with SoundMexPro toolbox (Hoertech, Germany) in MATLAB (R2018b, MathWorks, United States). For the playback experiment, echolocation calls were recorded and simulated echoes were played at a sampling rate of 1 MHz with custom-written LabVIEW programs with FPGA chips (PXIe-7858R, National Instruments, Austin, TX, United States).

### Data analysis

#### Sound analysis

Echolocation calls were batch-processed with custom-written scripts in MATLAB. The analysis scripts were created and tested in an earlier study (Lu et al., 2020). Before signal parameter estimation, sound recordings were bandpass filtered (“filtfilt” function) with 4th order Butterworth filter to keep the dominant second harmonic only. The filtered recording was rectified and smoothed (“smooth” function, with 25 points window size), from which background noise floor was estimated. Subsequently, the amplitude threshold for detecting calls was set 2–4 times of this noise floor based on the signal-to-noise (SNR) of the calls. For each identified call, we estimated a set of acoustic parameters, including the peak CF (call frequency of the maximum energy), peak call amplitude, call duration, and inter-pulse-interval (IPI), which are relevant to the current study. Peak CF was measured from an FFT size of 8,192, resulting in a frequency resolution of 23.4 Hz. Call duration was defined as the time difference between call onset and offset, which were both measured as the time points of –30 dB below the maximum call amplitude. IPI was defined as the time difference between the onset of two consecutive calls. Quality of sound analysis was manually checked for randomly selected recordings as a routine by displaying the waveform, power spectrum, and spectrogram graphically. Particular attention has also been paid to calls of low SNR such as those from the final phase of approaching the landing platform (Figure 2B). Manual checking confirmed the high quality of sound analysis. For the free flight experiment where an array of microphones was used, signal parameters were measured from the center microphone (Figure 1B) that had the best SNR.

#### Flight speed

We reconstructed the 3D position of the flying bats at the time of call emission with the microphone array. The 3D location was determined by the triangulation method using the time-of-arrival differences (TOAD) between the microphones. TOADs were computed by cross-correlating the isolated FM component of the dominant 2nd harmonic of the call, as the existence of the CF component seriously affects the accuracy. The FM component was isolated by filtering out the CF component with an elliptic filter (“ellip” function) with the cutoff frequency set to 3 kHz below the peak CF of the call. We only reconstructed the 3D position of the bat when the FM target signal of enough SNR (>12 dB) can be found in at least five recording channels. After reconstructing the 3D position of the bat, we applied a cubic smoothing spline (“csaps” function, with p set to 0.99) for each of the *x*-, *y*-, and *z*-axis data to avoid abrupt position jumping due to limited positioning accuracy. From the smoothed flight trajectory (Figure 1B), we estimated the instantaneous flight speed of the bat. As suggested by previous studies, the 3D position from the acoustic localization method may cause up to 10 cm position error in some extreme cases (Surlykke and Kalko, 2008; Surlykke et al., 2009). We found that positioning error particularly affects flight speed estimation when the bat is accelerating and deaccelerating which occurs at the beginning and end of a flight trial. Furthermore, we did not measure the actual landing time when bat touched the landing grid. Hence, we considered the flight speeds at the begging and end of a flight trial inaccurate and did not use them for further analysis.

#### Pendulum speed

We tracked the two-dimensional (2D) position of the two illuminated LEDs fixed to the side of the pendulum holder during the swings with custom-written scripts in MATLAB. The central positions of the two LEDs were located and their distance in pixel was measured for each frame. Because the physical distance between the two LEDs was fixed (15.3 cm), a change in the pixel distance signifies distortions of the camera lens. We computed the 2D positions of the LED after correcting camera lens distortion. The mid-point of the two LEDs was used to represent the pendulum, from which we estimated the pendulum speed.

#### Doppler shift compensation performance

We evaluated the DSC performance of *H. pratti* in the free flight and moving pendulum experiments quantitatively, but did not perform a detailed analysis of audiovocal adjustments in the playback experiment. This was because in the playback experiment *H. pratti* exhibited bidirectional adjustments of call frequency and the frequency adjustments were highly variable across the perturbations (Figures 4H,L), which were not consistent with DSC of call emissions to stabilize echo frequency. For each trial of the free flight experiment, we first located the “vocal” landing time which was defined as the time when *H. pratti* reached the maximum call rate, minimum call amplitude, or minimum call duration. Using the median of these landing time estimates as a reference, we evaluated DSC performance for the flight period of –1.2∼–0.2 s (Figure 5A). For the moving pendulum experiment, DSC performance was evaluated for the forward swing of the first cycle after correcting for the reaction time (Figure 5B). DSD performance was evaluated by two parameters, maximum compensation in percentage and DSC precision. Maximum compensation referred to the percentage ratio of the maximum frequency change in flight or in the forward swing (Figures 5A,B, red circles) to the expected Doppler effect. Compensation precision referred to the percentage ratio of the standard deviation to the mean of the reference frequency (echo frequency).

To calculate the emitted frequency of the bat during flight, we used the following equation:


Fs=Fm×(c−vb)/c.

where Fm is the signal frequency recorded by the ground microphone, vb is the flight speed of the bat relative to the wall directly to its front (i.e., the microphone wall), c is sound speed in air (343 m/s). Thus, we assumed that the flying bat performed the DSC using echoes from the microphone wall. This assumption was probably not valid during the final landing period when the bat rotates its body and head from a flight posture to a hanging posture, which was one of the reasons we excluded data from the landing maneuver in the DSC analyses (see Figure 5A).

From the emitted frequency, we further calculate the echo frequencies received by *H. pratti* during flight and in moving pendulum with the following equation:


$$|\bar{F_{e\,c\,h\,o}=F_{s}+F_{s}\times 2\times v_{b}/c}$$

Both equations were originally used Schnitzler (1973) and are commonly used for analyzing the call frequency of CF-FM bats (Schnitzler and Denzinger, 2011; Hiryu et al., 2016).

#### Statistical analysis

Statistical analyses were conducted to compare the DSC performance of *H. pratti* between the free flight and moving pendulum experiments, with the Statistical and Machine Learning toolbox of MATLAB. For all statistical tests, we used the non-parametric Wilcoxon signed-rank test (“ranksum” function) and Wilcoxon rank-sum test (“ranksum” function) test the difference between the medians for paired and non-paired comparisons respectively. A *P*-value of 0.05 was adopted to indicate a statistical significance. Statistical analyses were based on a total of 142 trials from four *H. pratti* in the free flight experiment, ranging from 34 to 36 trials across individual animals, and a total of 75 trials from four *H. pratti* in the moving pendulum experiment, ranging from 18 to 20 trials across individual animals. The total calls involved in the moving pendulum and free flight experiments were 33, 480 and 17, 074.

## Data availability statement

The raw data supporting the conclusions of this article will be made available by the authors, without undue reservation.

## Ethics statement

The animal study was reviewed and approved by the Institutional Animal Care and Use Committee of the Central China Normal University.

## Author contributions

ML, XW, and HW collected the data. JL, ML, and XW analyzed the data. JL and CM wrote the manuscript. All authors contributed to the article and approved the submitted version.
