# Corrigendum: Doppler shift compensation performance in *Hipposideros pratti* across experimental paradigms

**DOI:** 10.3389/fnsys.2025.1595428

**Published:** 2025-04-11

**Authors:** Jinhong Luo, Manman Lu, Xindong Wang, Huimin Wang, Cynthia F. Moss

**Affiliations:** ^1^School of Life Sciences, Institute of Evolution and Ecology, Central China Normal University, Wuhan, China; ^2^Department of Psychological and Brain Sciences, Johns Hopkins University, Baltimore, MD, United States

**Keywords:** auditory feedback, behavioral plasticity, echolocation, motor control, neuroethology

In the published article, there was an error in **Materials and methods**, subheading “Data Analysis,” *Doppler shift compensation performance*, paragraph 2, in which the plus symbol (+) should have instead been the multiply symbol (×) in the included equation.

The corrected equation appears below:

“[*F*_*s*_ = *F*_*m*_ × (*c* − *v*_*b*_)/*c*]”.

This is because the calculations were indeed based on the correct equation.

The authors apologize for this error and state that this does not change the scientific conclusions of the article in any way. The original article has been updated.

